# Prognostic Value of Neutrophil, Monocyte, Lymphocyte, and Platelet/High-Density Lipoprotein Ratios in Ischemic Heart Disease: An NHANES Analysis

**DOI:** 10.3390/medicina60122084

**Published:** 2024-12-19

**Authors:** Chia-Chen Wu, Chia-Hui Wu, Chien-Ho Lee, Tien-Yu Chen, Cheng-I Cheng

**Affiliations:** 1 Division of Thoracic and Cardiovascular Surgery, Department of Surgery, Kaohsiung Chang Gung Memorial Hospital, Chang Gung University College of Medicine, Kaohsiung City 833, Taiwan; maxwu02@gmail.com; 2 Department of Medical Imaging, Kaohsiung Medical University Chung-Ho Memorial Hospital, Kaohsiung City 807, Taiwan; chiaheuy@hotmail.com; 3 Division of Cardiology, Department of Internal Medicine, Kaohsiung Chang Gung Memorial Hospital, Chang Gung University College of Medicine, Kaohsiung City 833, Taiwan; gentolata@hotmail.com (C.-H.L.); a_den0904@hotmail.com (T.-Y.C.)

**Keywords:** ischemic heart disease (IHD), high-density lipoprotein (HDL), neutrophil/HDL ratio, lymphocyte/HDL ratio, monocyte/HDL ratio, mortality, national health and nutrition examination survey (NHANES)

## Abstract

The prognostic value of easily accessible hematologic biomarkers, such as the neutrophil-to-HDL ratio, as well as the monocyte-to-HDL, lymphocyte-to-HDL, and platelet-to-HDL ratios, remains underexplored in patients with established ischemic heart disease (IHD). Community-dwelling adults aged ≥ 20 with established IHD from the National Health and Nutrition Examination Survey (NHANES) 1999–2018 were eligible. Mortality was tracked through linkage to the National Death Index (NDI) until the end of 2019. Cox regressions examined the associations between these hematologic ratios and all-cause mortality. Receiver operating characteristic (ROC) curve analysis assessed the predictive accuracy of these ratios for mortality. A total of 2265 patients were analyzed, with a median follow-up period of 80 months. After adjusting for demographic factors, lifestyle variables, and comorbidities, patients in the highest quartile of the neutrophil/HDL had a significantly increased all-cause mortality risk (aHR = 1.41, 95% CI: 1.13–1.77) compared to those in the lowest quartile. No significant associations were found between the other ratios and mortality. In conclusion, this study found that among the hematologic ratios analyzed, an elevated neutrophil-to-HDL ratio has the strongest potential for mortality risk stratification in community-dwelling patients with ischemic heart disease in the US, offering important guidance for both patients and clinicians.

## 1. Introduction

Ischemic heart disease (IHD) remains a leading cause of illness and death worldwide, despite significant advances in its diagnosis and treatment [1]. In the United States (US), IHD—largely driven by coronary artery disease (CAD)—is responsible for approximately 610,000 deaths annually, accounting for nearly 1 in 4 fatalities. Globally, IHD ranks as the third leading cause of death, contributing to an estimated 17.8 million deaths each year [2]. CAD, the primary underlying cause of IHD, involves the buildup of atherosclerotic plaque in the coronary arteries, leading to reduced blood flow and an increased risk of myocardial infarction, angina, and other cardiovascular events [3]. Current pharmacological treatments, including antiplatelet agents, angiotensin-converting enzyme (ACE) inhibitors, angiotensin II receptor blockers (ARBs), beta blockers, and lipid-lowering drugs, are critical for the secondary prevention of IHD [4]. However, residual risk remains, underscoring the need for additional strategies to improve management and outcomes in patients with IHD.

In the era of precision medicine, there is increasing interest in novel biomarkers and risk prediction models to improve understanding and prognostication of CAD and IHD and the subsequent clinical outcomes [5,6,7]. Multiple studies have established the link between lipid profile and the progression and outcomes of IHD [8,9]. Large epidemiological studies have correlated serum high-density lipoprotein cholesterol (HDL) levels derived from routine blood biochemistry to mortality risk from IHD [10,11]. Furthermore, research suggests combining different lipid parameters, such as the total cholesterol (TC)/HDL ratio, for evaluating cardiovascular risk [12,13]. Nonetheless, there is a shortage of thorough assessment regarding its predictive importance in relation to the long-term mortality of non-institutionalized individuals.

Among the numerous hematologic biomarkers and ratios under scrutiny as potential predictors, the neutrophil/HDL, lymphocyte/HDL, monocyte/HDL, and platelet/HDL ratios have garnered interest due to their suspected associations with adverse health conditions, encompassing metabolic disorders, thrombotic diseases, and the prognosis of acute coronary syndrome [14,15,16,17]. They are viewed as potential markers of systemic inflammation and oxidative stress in inflammatory conditions, reflecting the complex interaction between lipid metabolism, immune response, and atherogenesis [18]. However, previous studies have primarily focused on either acute hospital settings or healthy populations, with none specifically examining individuals with IHD living in the community. As a result, these biomarkers offer intriguing possibilities for risk stratification in patients living with IHD, though research on their predictive value for long-term mortality in this population remains limited.

Understanding these biomarkers’ prognostic significance can improve risk assessment, help identify high-risk individuals, and may guide tailored care protocols for individuals living with IHD. Hence, this research leverages the extensive data from the US National Health and Nutrition Examination Survey (NHANES) to investigate the prognostic value of neutrophil/HDL, platelet/HDL, monocyte/HDL, and lymphocyte/HDL ratios, as well as the TC/HDL ratio, in community-residing individuals with established IHD diagnoses.

## 2. Methods

### 2.1. Data Source and Study Design

This retrospective cohort study was based on data from the NHANES, overseen by the National Center for Health Statistics (NCHS), a division of the US Centers for Disease Control and Prevention (CDC). NHANES is a critical program that integrates interviews, physical examinations, and laboratory measurements to monitor the health and nutritional statuses of individuals across the US. Initially, participants provide information during in-home interviews and then undergo detailed assessments at a mobile examination center, including physical exams, measurements, and lab tests. The NAHNES program employs a detailed and complex design to ensure the data accurately reflects the broader, non-institutionalized US population.

### 2.2. Ethical Statement

The NHANES program is rigorously reviewed and approved by the NCHS Research Ethics Review Board (ERB). Participants in the survey sign an informed consent form prior to participation, with the protocol number and approval details available on the NHANES website at https://www.cdc.gov/nchs/nhanes/about/erb.html (accessed on 1 February 2024). All data from NHANES are anonymized. As a result, no further ethical approval or additional consent was required for analysis.

### 2.3. Participants

NHANES participants aged 20 or older with established IHD were eligible for inclusion, with data collected over eight NHANES dual-year cycles from 1999 to 2018. IHD was confirmed when participants reported ever having a physician’s diagnosis of any of the following conditions: coronary heart disease, angina, or myocardial infarction (MI). Participants who reported heart failure on the questionnaire were excluded from the study cohort, as heart failure is often a consequence of IHD, is strongly associated with mortality, and could potentially obscure the associations under investigation. Participants were also excluded if they lacked mortality status during follow-up or had incomplete data on the primary study variables.

### 2.4. Mortality Data

The primary endpoint was all-cause mortality, tracking deaths from any source via linkage with the National Death Index (NDI), which provides updated death records through 2019. The NDI is a centralized database of death record information that helps researchers access up-to-date mortality data. Further information about the data linkage process and access to mortality data can be found at the following CDC link: https://www.cdc.gov/nchs/data-linkage/mortality-public.htm (accessed on 1 February 2024).

### 2.5. Neutrophil, Monocyte, Lymphocyte, Platelet, and TC/HDL Ratios

The hematologic ratios of interest were derived from the complete blood count (CBC) and standard biochemistry profile provided by NHANES. Blood samples from each participant were collected at the time of entry into the NHANES survey. The CBC parameters were measured and analyzed using the Beckman Coulter method, which includes automated counting, sizing, diluting, and mixing, along with hemoglobin measurement by a single-beam photometer. The WBC differential employed Volume, Conductivity, and Scatter (VCS) technology. TC and HDL levels were measured from blood samples collected during the study using an enzymatic assay detailed in the NHANES Laboratory/Medical Technologists Procedures Manual. This assay involves converting esterified to free cholesterol, then using the Trinder reaction for a colorimetric readout at 505 nm, a method specific to cholesterol testing (https://wwwn.cdc.gov/Nchs/Data/Nhanes/Public/2011/DataFiles/TCHOL_G.htm (accessed on 1 February 2024)). We evaluated the associations between the neutrophil, lymphocyte, monocyte, and platelet/HDL ratios, as well as the TC/HDL ratio, with all-cause mortality. These biomarkers were analyzed both as continuous variables (using z-scores) and in quartiles.

### 2.6. Other Variables

Information on age, sex, race, income-to-poverty ratio, marital status, and education level was collected through face-to-face interviews conducted by skilled interviewers. The body mass index (BMI) was derived from measurements taken during NHANES examinations.

Participants were categorized based on smoking history: those who smoked fewer than 100 cigarettes in their lifetime as non-smokers; those who smoked more than 100 cigarettes but had quit as former smokers; and those who smoked over 100 cigarettes and currently smoked as current smokers.

Systolic (SBP) and diastolic blood pressure (DBP) were measured three times using a standardized protocol and averaged for analysis. Hypertension was defined as a “yes” response to being told on multiple visits about having hypertension, being advised to take medication for it, or having an average SBP ≥ 140 mmHg or DBP ≥ 90 mmHg from three readings.

Participants diagnosed with diabetes mellitus (DM) were excluded if they met any of the following criteria: doctor’s diagnosis, use of blood-sugar-lowering medication, insulin usage or laboratory results showing HbA1c ≥ 6.5%, fasting glucose ≥ 126 mg/dL, or glucose ≥ 200 mg/dL during an oral glucose tolerance test (OGTT).

The glomerular filtration rate (GFR) was calculated using the 4-variable Modification of Diet in Renal Disease (MDRD) Study equation, based on recalibrated serum creatinine levels. In this instance, we utilized the IDMS-traceable MDRD Study equation that employs standardized creatinine: GFR = 175 × (standardized serum creatinine) − 1.154 × (age) − 0.203 × 0.742 (for female) × 1.212 (for African American), presented in ml/min/1.73 m^2^. Chronic kidney disease (CKD) was defined by having an eGFR < 60 ml/min/1.73 m^2^.

Chronic respiratory disease was determined based on participants’ self-reported diagnoses of asthma, chronic bronchitis, or emphysema. Similarly, histories of stroke, cancer, and the time since being diagnosed with CAD were identified using the same method.

Medication use data, including beta blockers, ACE inhibitors/ARBs, and statins, were obtained from NHANES’ prescription medication file. Participants reported any prescription medications used in the past 30 days during household interviews and presented medication containers; however, dosage information was not collected. Further details are available on the NHANES website at the following link: https://wwwn.cdc.gov/Nchs/Data/Nhanes/Public/2003/DataFiles/RXQ_RX_C.htm (accessed on 1 February 2024).

### 2.7. Statistical Analysis

To guarantee national representation, specific sample weights (WTSAF2YR), strata (SDMVSTRA), and clusters (SDMVPSU) were utilized. Continuous variables are displayed as mean ± standard deviation (SD) for demographic information and median (interquartile range, IQR) for medical history and laboratory data, while categorical variables are shown as n (%). Throughout the study’s follow-up period, differences between patients who died and those who did not were assessed using the Wilcoxon rank sum test for continuous data and Pearson’s Chi-squared test for categorical data. Both univariate and multivariable Cox regression analyses were conducted to explore the relationships between all-cause mortality and the biomarkers. The findings are reported as both crude and adjusted hazard ratios (HRs) with their respective 95% confidence intervals (CIs).

A receiver operating characteristics (ROC) curve analysis was performed to assess the effectiveness of the markers in predicting all-cause mortality, indicated by the area under the ROC curve (AUC) along with the corresponding 95% CI. All *p*-values are two-sided, with a value of *p* < 0.05 considered significant. Data management and analyses were conducted using R studio software (https://www.r-project.org/, accessed on 5 November 2024).

## 3. Results

### 3.1. Patient Selection

After excluding patients with heart failure, without mortality status at follow-up, or complete information on the indices of interest, 2265 adults with established IHD were identified and included in the analysis.

### 3.2. Characteristics of the Study Participants

The characteristics of the study participants are summarized in Table 1. During a median follow-up time of 80 months, there were a total of 844 deaths. Participants who died during follow-up were older than those who survived (72.8 vs. 62.7, *p* < 0.001). Distribution of sex, race, BMI, poverty income ratio, education level, and smoking status were significantly different between individuals who were alive or deceased (*p* = 0.04 to <0.001). Concerning comorbid conditions, hypertension, chronic respiratory disease, history of stroke, history of cancer, and CKD were differently distributed between the two groups (*p* = 0.006 to <0.001). Participants who were alive had a larger proportion of statin users (58% vs. 53%, *p* = 0.028). With regard to laboratory measures, serum total cholesterol, neutrophil, monocyte, and lymphocyte counts were significantly different between patients who died and survived (*p* = 0.043 to <0.001) (Table 1).

Table 2 summarizes the biomarkers’ levels by mortality status. Among the biomarkers, neutrophil/HDL (*p* = 0.01) and lymphocyte/HDL ratios (*p* < 0.001) were significantly different between participants who died and survived, whereas the others showed no significant between-group difference (Table 2).

### 3.3. Associations Between the Hematologic Ratios and Mortality in Patients with IHD

Associations between the hematologic ratios and all-cause mortality in community-dwelling patients with IHD are summarized in Table 3. When treated as continuous variables, after adjusting for age (in years), sex, race, DM, history of cancer, chronic respiratory disease, and CKD in multivariable model 1, per unit increase in neutrophil/HDL ratio (z-score) was significantly associated with increased risk of all-cause mortality (aHR = 1.18, 95%CI: 1.10–1.28). Also, the highest quartile (Q4) of neutrophil/HDL ratio was significantly associated with a higher risk of mortality (aHR = 1.44, 95%CI: 1.17–1.76) as compared to the lowest quartile (Q1).

Moreover, after further adjusting for education level, poverty income ratio, BMI, year since diagnosis of CAD, hypertension, history of stroke, and medication usage in multivariable model 2, neutrophil/HDL ratio remained significantly associated with increased mortality risk. This was observed both as a continuous variable (aHR = 1.17, 95%CI: 1.08–1.28) and when comparing quartiles (Q4 vs. Q1: aHR = 1.41, 95%CI: 1.13–1.77). No significant associations were observed between all-cause mortality risk and monocyte/HDL, platelet/HDL, lymphocyte/HDL, or TC/HDL ratios (Table 3).

**Figure 1:**
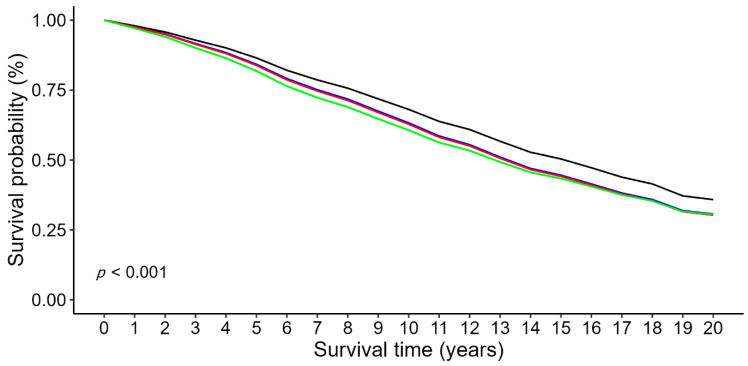
Mean predicted survival probability in patients with IHD across different levels of neutrophil/HDL ratio.

Mean predicted survival probability for patients across neutrophil/HDL ratio quartiles is plotted in Figure 1.

### 3.4. ROC Curve Analysis of the Hematologic Ratios in Predicting Mortality in Patients with IHD

**Figure 2:**
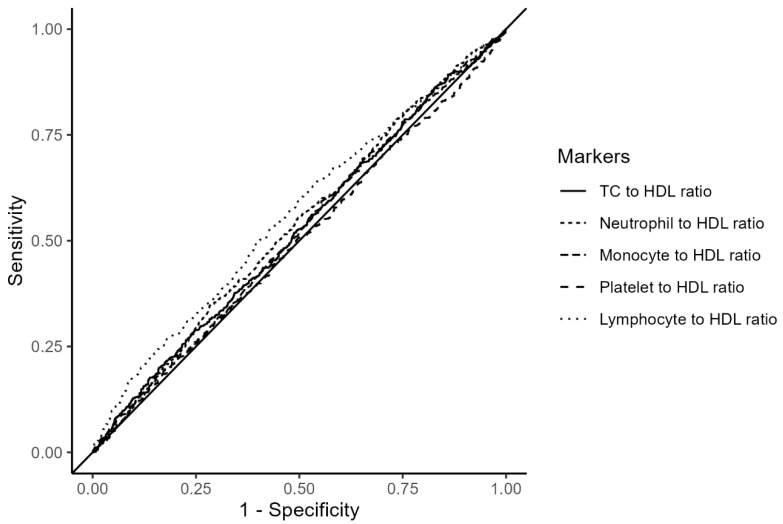
ROC curves of the hematologic ratios for predicting all-cause mortality in patients with IHD. Abbreviations: IHD, ischemic heart disease; Q, quartile; ROC, receiver-operating characteristics.

The predictive performance of the hematologic ratios for all-cause mortality is shown in Figure 2 and Supplementary Table S1. The AUCs of TC/HDL, neutrophil/HDL, monocyte/HDL, platelet/HDL, and lymphocyte/HDL ratios were 0.52 (95%CI: 0.50–0.55), 0.53 (95%CI: 0.51–0.56), 0.52 (95%CI: 0.49–0.54), 0.50 (95%CI: 0.47–0.52), and 0.56 (0.54–0.59), respectively (Figure 2 and Supplementary Table S1).

## 4. Discussion

This study represents the first comprehensive investigation into the relationships between neutrophil/HDL, monocyte/HDL, platelet/HDL, lymphocyte/HDL, and TC/HDL ratios and long-term all-cause mortality among community-dwelling individuals with established IHD in the US. After adjusting for relevant demographic factors, lifestyle habits, and baseline comorbidities, our findings indicate a distinct and independent association between a high neutrophil-to-HDL ratio and an approximately 40% increased risk of all-cause mortality. Despite the observed association, the predictive power of these ratios, as reflected by the AUC values ranging from 0.50 to 0.56, suggests poor performance as standalone predictors [19]. In summary, the neutrophil/HDL ratio emerges as a potential marker for mortality risk stratification in patients living with IHD. Further research is needed to enhance its predictive utility and explore its integration with other clinical factors.

Chronic inflammation and disrupted lipid metabolism are key contributors to atherosclerosis and its associated cardiovascular events and mortality [20]. Combinations of lipid metrics have been investigated for improved predictive accuracy. A previous study demonstrated that a high TC/HDL ratio is associated with an increased risk of atherosclerotic cardiovascular disease, independent of clinical risk factors [21]. Zhou et al. found that a high TC/HDL ratio in a healthy population was associated with increased cardiovascular mortality [13]. Our study on established IHD patients differs from prior research linking the TC/HDL ratio to adverse outcomes in general populations. The findings do not support an independent association between a high TC/HDL ratio and mortality, highlighting the importance of selecting prognostic markers tailored specifically for this population. This disparity may be explained by changes in HDL functionality during acute MI and reperfusion, where HDL, normally cardioprotective, becomes oxidized, captures proinflammatory molecules, and loses its beneficial properties [22,23]. In advanced IHD cases, factors other than lipids might have a more dominant influence on mortality. Also, following an IHD diagnosis, patients may implement lifestyle modifications or adhere to stringent medical regimens that can shift the prognostic value of traditional biomarkers. 

Our major finding is that the neutrophil/HDL ratio’s association with mortality surpasses other hematologic ratios and aligns with existing literature. Hematologic indicators, such as neutrophil and lymphocyte counts combined with HDL ratios, have recently been evaluated as cost-effective markers of inflammation and oxidative stress [15,24,25]. While not extensively documented in medical literature, several studies have assessed the prognostic significance of these markers concerning the development and progression of IHD. Pan et al. [26] highlighted a significant correlation between neutrophil/HDL ratio and cardiac ultrasound parameters, indicating its association with cardiovascular risk in healthy populations. In a study of older Chinese patients with acute MI, Huang et al. identified the neutrophil/HDL ratio as the only significant predictor of long-term mortality and recurrent MI after multivariable adjustment [14]. Ozgeyik et al. found that the neutrophil/HDL ratio predicts long-term prognosis in Turkish patients with total coronary artery occlusion and outperforms other ratios, such as monocyte/HDL, TG/HDL, HDL/LDL, and lymphocyte/HDL, in predicting mortality [27]. A recent review by Lamichhane et al. [28] highlighted the neutrophil/HDL ratio’s strong sensitivity and moderate specificity for detecting significant coronary stenosis, outperforming traditional parameters. It also shows promise for stratifying risk in acute coronary syndrome, including long-term mortality. Jiang et al. identified it as a predictor of long-term outcomes but did not focus on IHD patients or compare it with other hematologic marker-to-HDL ratios [29].

Additionally, Liu et al. [30] demonstrated that an elevated neutrophil/HDL ratio was independently associated with increased cardiovascular event risk in normoglycemic individuals. Similarly, Guo et al. [31] reported the clinical utility of neutrophil/HDL as a predictive indicator in STEMI patients undergoing percutaneous coronary intervention, showing its relevance for both major adverse cardiovascular events (MACE) during hospitalization and the degree of coronary artery stenosis. Moreover, a recent study by Gao et al. [32] emphasized that neutrophil/HDL was not only associated with the presence and severity of CAD but also outperformed markers like neutrophil count, HDL-C, or LDL-C/HDL-C in predicting severe CAD. Collectively, these findings, along with our results, underscore the potential of the neutrophil/HDL ratio as a valuable prognostic indicator in various cardiovascular contexts.

Although an association was observed, the mechanisms underlying the relationship between the neutrophil/HDL ratio and mortality in IHD patients cannot be fully clarified by this study; however, prior experimental studies may offer insights into the potential biological mechanisms. Neutrophils, constituting a significant segment of white blood cells, have been acknowledged for their involvement throughout multiple stages of atherosclerosis [33]. They exacerbate endothelial dysfunction, recruit monocytes to atherosclerotic sites, activate macrophages, promote foam cell formation, destabilize plaques, and act as frontline responders in inflammation by activating monocytes and lymphocytes [33]. On the other hand, HDL is protective against atherosclerosis, primarily through reverse cholesterol transport, antioxidant properties, and anti-inflammatory effects on the endothelium [34]. HDL, enriched in lipid rafts, inhibits neutrophil activity, promotes endothelial angiogenesis, and prevents cardiomyocyte apoptosis, supporting its cardioprotective role [34]. Therefore, this collectively explains why combining neutrophils and HDL is expected to provide enhanced prognostic value compared to their standalone use.

In summary, our study identifies the neutrophil/HDL ratio as an independent prognostic biomarker for all-cause mortality in patients with established IHD, supported by the mechanistic insights discussed above. However, the overall modest predictive performance of this ratio indicates the need for more nuanced research approaches before they can be adopted in clinical practice. Future studies should consider controlling for factors such as IHD severity, thereby enhancing the precision of mortality predictions and informing more effective clinical interventions.

### Strengths and Limitations

This study’s significant advantage lies in its use of data from NHANES, sourced from a broad and varied segment of the US populace, ensuring that its results are applicable across the entire nation. Moreover, the extended duration of follow-up further strengthens this study. The analyses were rigorously adjusted for demographic characteristics, lifestyle factors, medication use, and comorbid conditions, enhancing their reliability. Nonetheless, this study does have several limitations. Firstly, as this is a community-based study rather than a hospital-based one, it lacks direct diagnostic confirmation of IHD, such as coronary angiography. Consequently, the severity of IHD based on imaging or other diagnostic evaluations was not considered. While the duration of CAD was considered and included, the details relied on participant-reported questionnaires, potentially introducing recall bias. Although this study considered several key medications, including ACEIs/ARBs and statins, it did not account for antithrombotic therapy or coronary interventions due to the lack of available data. Moreover, the hematologic ratios were assessed only at baseline and were thus not treated as time-dependent variables, potentially introducing bias, as they could dynamically change over the follow-up period. Another important limitation is the lack of external validation, which is crucial for confirming the robustness and generalizability of our model and should be addressed in future research. Lastly, although a statistical association between the neutrophil/HDL ratio and mortality exists, its predictive ability assessed by ROC curve analysis does not significantly exceed or improve upon that of existing predictive tools, limiting its immediate applicability. For more precise prognostication, future research should include the severity of IHD, as well as medications and intervention profiles. Another consideration is to explore whether combining these markers or integrating them with additional clinical parameters could enhance prognostic accuracy. Lastly, research should focus on longitudinal studies that monitor biomarker levels and track disease progression over time to yield insights into the dynamic nature of these biomarkers and their correlation with the pathophysiology of IHD.

## 5. Conclusions

Among the hematologic ratios analyzed, the neutrophil/HDL ratio is independently associated with all-cause mortality in community-dwelling IHD patients. Other indices, such as TC/HDL, monocyte/HDL, platelet/HDL, and lymphocyte/HDL ratios, do not demonstrate significant associations with mortality. Future research should account for the severity of IHD, along with considerations of medication use and surgical history, to improve its predictive accuracy.

## Figures and Tables

**Table 1:** Characteristics of patients with IHD by mortality status.

Characteristics	Overall, N = 2265	Mortality Status		*p*-Value
		Alive, N = 1421	Deceased, N = 844	
Age, years	66.5 ± 12.7	62.7 ± 12.9	72.8 ± 9.5	**<0.001**
Age, category				**<0.001**
20–49	242 (11%)	220 (15%)	22 (2.6%)	
50–59	318 (14%)	255 (18%)	63 (7.5%)	
60–69	642 (28%)	467 (33%)	175 (21%)	
70–79	610 (27%)	315 (22%)	295 (35%)	
80+	453 (20%)	164 (12%)	289 (34%)	
Sex				**0.018**
Male	1370 (60%)	833 (59%)	537 (64%)	
Female	895 (40%)	588 (41%)	307 (36%)	
Race				**<0.001**
Non-Hispanic White	1311 (58%)	734 (52%)	577 (68%)	
Non-Hispanic Black	366 (16%)	249 (18%)	117 (14%)	
Hispanic White	162 (7.2%)	132 (9.3%)	30 (3.6%)	
Other/Unknown	426 (19%)	306 (22%)	120 (14%)	
BMI, kg/m^2^	29.5(6.4)	30.3 ± 6.7)	28.1 ± 5.7	**<0.001**
BMI, category				**<0.001**
<25.0	543 (24%)	284 (20%)	259 (31%)	
25.0–29.9	812 (36%)	501 (35%)	311 (37%)	
≥30	910 (40%)	636 (45%)	274 (32%)	
Years since CAD diagnosed	11.4 ± 10.9	11.2 ± 10.7	11.5 ± 11.2	0.602
<5 years	718 (32%)	457 (32%)	261 (32%)	
≥5 years	1516 (68%)	952 (68%)	564 (68%)	0.697
Poverty income ratio				**0.04**
>1	1602 (78%)	980 (76%)	622 (80%)	
≤1	456 (22%)	303 (24%)	153 (20%)	
Education level				**<0.001**
Above high school	1527 (68%)	1015 (72%)	512 (61%)	
Never attended high school	732 (32%)	404 (28%)	328 (39%)	
Smoking status				**<0.001**
Never	871 (38%)	575 (40%)	296 (35%)	
Former	950 (42%)	550 (39%)	400 (47%)	
Current	444 (20%)	296 (21%)	148 (18%)	
DM	804 (35%)	485 (34%)	319 (38%)	0.078
Hypertension	1670 (75%)	1014 (72%)	656 (79%)	**<0.001**
SBP, mmHg	113.6 ± 22.0	130.5 ± 20.1	138.9 ± 24.0	**<0.001**
DBP, mmHg	67.4 ± 15.5	69.2 ± 14.3	64.3 ± 16.9	**<0.001**
Chronic respiratory disease	604 (27%)	411 (29%)	193 (23%)	**0.002**
History of stroke	306 (14%)	166 (12%)	140 (17%)	**<0.001**
History of cancer	463 (20%)	265 (19%)	198 (23%)	**0.006**
CKD	633 (28%)	320 (23%)	313 (37%)	**<0.001**
Medication				
Beta Blocker	1055 (47%)	658 (46%)	397 (47%)	0.735
ACEI/ARB	1143 (50%)	733 (52%)	410 (49%)	0.167
Statin	1267 (56%)	820 (58%)	447 (53%)	**0.028**
HDL, md/dL	49.9 ± 15.4	49.6 ± 14.9	50.4 ± 16.2	0.713
Total Cholesterol, md/dL	184.5 ± 44.7	181.9 ± 43.3	188.7 ± 46.7	**0.005**
Neutrophil, 10^3^ cells/uL	4.4 ± 1.7	4.3 ± 1.7	4.6 ± 1.8	**<0.001**
Monocyte, 10^3^ cells/uL	0.6 ± 0.2	0.6 ± 0.2	0.6 ± 0.2	**0.043**
Platelet, 10^3^ cells/uL	232.1 ± 67.4	230.2 ± 64.1	235.3 ± 72.7	0.176
Lymphocyte, 10^3^ cells/uL	2.1 ± 2.9	2.1 ± 2.0	2.1 ± 4.0	**<0.001**
Survival times, months	80 (44,137)	85 (44,143)	75 (43,124.3)	**<0.001**

Continuous data are presented as mean ± standard deviation (SD) or median with interquartile range (IQR). Categorical data are presented as count and percentage. *p*-value was derived using the Wilcoxon rank sum test for continuous data and Pearson’s Chi-squared test for categorical data. *p*-value < 0.05 is shown in bold. ACEI, angiotensin-converting enzyme inhibitors; ARB, angiotensin receptor blockers; SD, standard deviation; BMI, body mass index; SBP, systolic blood pressure; DBP, diastolic blood pressure; CAD, coronary artery disease; CKD, chronic kidney disease; DM, diabetes mellitus; HDL, high-density lipoprotein cholesterol; IHD, ischemic heart disease.

**Table 2:** Neutrophil/HDL, monocyte/HDL, platelet/HDL, lymphocyte/HDL, and TC/HDL ratios of patients with IHD by mortality status.

Hematologic Indices	Overall, N = 2265	Mortality Status		*p*-Value
		Alive, N = 1421	Deceased, N = 844	
TC/HDL ratio, continuous	3.967 ± 1.392	3.930 ± 1.391	4.030 ± 1.393	0.082
TC/HDL ratio, quartiles				0.27
Q1	566 (25%)	365 (26%)	201 (24%)	
Q2	566 (25%)	361 (25%)	205 (24%)	
Q3	565 (25%)	358 (25%)	207 (25%)	
Q4	568 (25%)	337 (24%)	231 (27%)	
Neutrophil/HDL ratio, continuous	0.097 ± 0.050	0.096 ± 0.051	0.100 ± 0.050	**0.01**
Neutrophil/HDL ratio, quartiles				**0.036**
Q1	563 (25%)	379 (27%)	184 (22%)	
Q2	568 (25%)	355 (25%)	213 (25%)	
Q3	566 (25%)	352 (25%)	214 (25%)	
Q4	568 (25%)	335 (24%)	233 (28%)	
Monocyte/HDL ratio, continuous	0.013 ± 0.007	0.013 ± 0.007	0.014 ± 0.007	0.219
Monocyte/HDL ratio, quartiles				0.799
Q1	563 (25%)	363 (26%)	200 (24%)	
Q2	564 (25%)	352 (25%)	212 (25%)	
Q3	561 (25%)	347 (24%)	214 (25%)	
Q4	577 (25%)	359 (25%)	218 (26%)	
Platelet/HDL ratio, continuous	5.039 ± 2.058	5.012 ± 1.980	5.084 ± 2.183	0.9
Platelet/HDL ratio, quartiles				0.907
Q1	566 (25%)	349 (25%)	217 (26%)	
Q2	566 (25%)	356 (25%)	210 (25%)	
Q3	566 (25%)	361 (25%)	205 (24%)	
Q4	567 (25%)	355 (25%)	212 (25%)	
Lymphocyte/HDL ratio, continuous	0.046 ± 0.064	0.047 ± 0.047	0.046 ± 0.085	**<0.001**
Lymphocyte/HDL ratio, quartiles				**<0.001**
Q1	556 (25%)	310 (22%)	246 (29%)	
Q2	575 (25%)	352 (25%)	223 (26%)	
Q3	567 (25%)	377 (27%)	190 (23%)	
Q4	567 (25%)	382 (27%)	185 (22%)	

Continuous data are presented as mean ± SD. Categorical data are presented as count and percentage. *p*-value was derived using the Wilcoxon rank sum test for continuous data and Pearson’s Chi-squared test for categorical data. *p*-value < 0.05 is shown in bold. CAD, coronary artery disease; TC, total cholesterol; HDL, high-density lipoprotein cholesterol; SD, standard deviation; Q, quartile; IHD, ischemic heart disease.

**Table 3:** Univariate and multivariable Cox regression analysis for the association between the hematologic indices and all-cause mortality in patients with IHD.

Hematologic Indices	Univariate		Multivariable Model 1		Multivariable Model 2	
	Crude HR (95%CI)	*p*-Value	aHR (95%CI)	*p*-Value	aHR (95%CI)	*p*-Value
TC/HDL ratio, z-score	0.91 (0.84, 0.97)	**0.007**	1.07 (0.99, 1.15)	0.075	1.01 (0.93, 1.10)	0.8
Q1	Reference		Reference		Reference	
Q2	0.81 (0.67, 0.99)	**0.038**	0.89 (0.73, 1.08)	0.2	0.84 (0.68, 1.04)	0.11
Q3	0.74 (0.61, 0.90)	**0.002**	0.97 (0.79, 1.18)	0.7	0.9 (0.73, 1.12)	0.4
Q4	0.75 (0.62, 0.90)	**0.003**	1.13 (0.93, 1.38)	0.2	0.99 (0.79, 1.23)	0.9
Neutrophil/HDL ratio, z-score	1.09 (1.02, 1.16)	**0.015**	1.18 (1.10, 1.28)	**<0.001**	1.17 (1.08, 1.28)	**<0.001**
Q1	Reference		Reference		Reference	
Q2	1.06 (0.87, 1.29)	0.584	0.95 (0.78, 1.17)	0.7	0.99 (0.80, 1.24)	>0.9
Q3	1.12 (0.92, 1.36)	0.279	1.14 (0.93, 1.40)	0.2	1.14 (0.92, 1.43)	0.2
Q4	1.26 (1.04, 1.53)	**0.019**	1.44 (1.17, 1.76)	**<0.001**	1.41 (1.13, 1.77)	**0.003**
Monocyte/HDL ratio, z-score	1.08 (1.01, 1.16)	**0.025**	1.07 (0.99, 1.15)	0.081	1.06 (0.98, 1.15)	0.2
Q1	Reference		Reference		Reference	
Q2	1.04 (0.86, 1.26)	0.689	0.97 (0.79, 1.18)	0.7	0.96 (0.77, 1.19)	0.7
Q3	1.09 (0.90, 1.32)	0.389	1.14 (0.94, 1.40)	0.2	1.11 (0.90, 1.38)	0.3
Q4	1.08 (0.89, 1.30)	0.452	1.09 (0.89, 1.34)	0.4	1.04 (0.83, 1.30)	0.7
Platelet/HDL ratio, z-score	0.88 (0.82, 0.95)	**<0.001**	1.05 (0.97, 1.13)	0.2	1.03 (0.95, 1.12)	0.5
Q1	Reference		Reference		Reference	
Q2	0.81 (0.67, 0.98)	**0.031**	0.91 (0.75, 1.10)	0.3	0.89 (0.72, 1.10)	0.3
Q3	0.69 (0.57, 0.83)	**<0.001**	0.89 (0.73, 1.08)	0.3	0.83 (0.67, 1.03)	0.089
Q4	0.64 (0.53, 0.77)	**<0.001**	1.04 (0.85, 1.27)	0.7	0.99 (0.79, 1.23)	0.9
Lymphocyte/HDL ratio, z-score	0.99 (0.87, 1.13)	0.878	1.05 (1.00, 1.10)	0.05	1.05 (0.99, 1.10)	0.084
Q1	Reference		Reference		Reference	
Q2	0.83 (0.70, 1.00)	0.051	0.87 (0.73, 1.05)	0.14	0.91 (0.75, 1.11)	0.3
Q3	0.67 (0.56, 0.81)	**<0.001**	0.9 (0.74, 1.09)	0.3	0.82 (0.67, 1.01)	0.069
Q4	0.62 (0.51, 0.75)	**<0.001**	0.96 (0.78, 1.17)	0.7	0.91 (0.73, 1.13)	0.4

Multivariable model 1 adjusted for age (in years), sex, race, DM, history of cancer, chronic respiratory disease, and CKD. BMI, body mass index; DM, diabetes mellitus; CAD, coronary artery disease; CI, confidence interval; CKD, chronic kidney disease; HDL, high-density lipoprotein cholesterol; HR, hazard ratio; HR, adjusted HR; TC, total cholesterol; Q, quartile; IHD, ischemic heart disease. Multivariable model 2 adjusted for model 1 plus education, poverty income ratio, BMI category, year since diagnosis of CAD, hypertension, history of stroke, and medication use.

## Data Availability

All pertinent data are included within this manuscript.

## Supplementary Materials

The following supporting information can be downloaded at: https://www.mdpi.com/article/10.3390/medicina60122084/s1, Table S1: AUCs of different hematologic indices in predicting all-cause mortality in patients with IHD.
