# Supplementary material for: Prognostic Value of Neutrophil, Monocyte, Lymphocyte, and Platelet/High-Density Lipoprotein Ratios in Ischemic Heart Disease: An NHANES Analysis
Source: Medicina (Kaunas). 2024 Dec 19;60(12):2084. doi: 10.3390/medicina60122084 (PMC11678046; doi:10.3390/medicina60122084)
Supplement: Supplementary file 1 [file medicina-60-02084-s001.zip › medicina-3327829-supplementary.pdf]

Supplementary Table S1. AUCs of different hematologic indices in predicting all-cause mortality in patients with IHD.

Indices	AUC (95%CI)	Sensitivity	Specificity
Lymphocyte/HDL ratio	0.560 (0.536, 0.585)	99.8%	0.1%
Neutrophil/HDL ratio	0.532 (0.507, 0.557)	0%	99.9%
TC/HDL ratio	0.521 (0.497, 0.546)	100%	0%
Monocyte/HDL ratio	0.515 (0.491, 0.539)	0%	100%
Platelet/HDL ratio	0.498 (0.473, 0.523)	0%	100%

AUC, area under the curve; CI, confidence interval; CAD, coronary artery disease; TC, total cholesterol; HDL, high-density lipoprotein cholesterol; IHD, ischemic heart disease.
